# Young People’s Perspectives on Age Verification for Online Pornography in Australia: Cross-Sectional Online Survey

**DOI:** 10.2196/79622

**Published:** 2026-08-18

**Authors:** Jake Turvey, Sarah Eddy, Ana Orozco, Megan S C Lim

**Affiliations:** 1 Burnet Institute, 85 Commercial Rd, Melbourne, 3004, Australia, 61 392822111; 2 School of Population Health and Preventive Medicine, Monash University, Melbourne, Australia; 3 Melbourne School of Global and Population Health, The University of Melbourne, Parkville, Australia

**Keywords:** pornography, online safety, adolescents, survey, policy

## Abstract

**Background:**

Policymakers are increasingly seeking to reduce the accessibility of internet-based pornography to children and young people. In 2026, the Australian government implemented mandatory age verification for access to pornography and some other online content.

**Objective:**

This study aimed to understand young people’s attitudes toward age verification, including which methods of age verification they considered acceptable, and to explore associations between these attitudes and participant characteristics.

**Methods:**

A cross-sectional online survey of young people aged 15 to 29 years in Victoria, Australia (N=794) was conducted through social media advertising. Participants were asked whether they supported age verification for pornography and which of the 4 potential verification methods they considered acceptable. They could provide feedback about age verification in a free-text response. Logistic regression examined associations between participant characteristics and support for age verification (strongly agree or agree vs strongly disagree or disagree). A qualitative analysis of free-text responses was undertaken using a reflexive thematic analysis.

**Results:**

Of 761 respondents, most (n=459, 60.3%) supported mandatory age verification, but none of the 4 proposed specific age verification methods (identification document upload, facial scanning, credit card, and parental monitoring) was considered acceptable by a majority. The median age at which participants thought viewing pornography should be permitted was 16 (IQR 15-18) years. Those identifying as women, aged 18 to 19 years, actively religious, and who first intentionally viewed pornography at an older age were more likely to support age verification, whereas those identifying as LGBTQIA+ (lesbian, gay, bisexual, transgender, queer, intersex, asexual, and other sexual and gender minority identities), in an open relationship, and who viewed pornography monthly or more often were less likely to support it. Qualitative analysis of open-ended questions indicated that participants had considerable apprehensions related to user privacy and data security, potential adverse effects of blocking access to pornography on young people’s sexual development, and doubts about the overall effectiveness of the technology. Less regulatory strategies, such as comprehensive sexual health education, were discussed as more suitable and viable approaches.

**Conclusions:**

Policy changes are more likely to be successful if they have popular support. Although many young people in this study supported age verification as a concept, there were significant concerns about the feasibility and security of the process. It is vital to conduct further research on user apprehensions and the potential negative impacts of age verification and to explore more suitable, privacy-sensitive approaches before these policies are implemented.

## Introduction

As internet-based pornography becomes increasingly prevalent and accessible, children and young people in Australia are encountering sexually explicit content online with greater frequency and ease. An estimated 44% of Australian children aged between 9 and 16 years have encountered online pornography within the past month [1]. The median age at which young people first intentionally view pornography is 13 (IQR 12-15) years, although many are accidentally exposed to sexually explicit material at younger ages [2]. Early and unregulated exposure to pornographic content has been associated with harmful outcomes in young children, including impacts on sexual maturation and behavior [1-3]. Pornography use has been associated with an earlier age at first sexual experience, condomless sex, or sex with multiple partners, as well as sexist attitudes and attitudes supporting violence against women [2,4-6]. On the other hand, curiosity about sexuality is normal for young people, and many report pleasurable and positive experiences related to exploring pornography [7,8].

Policymakers have been seeking new ways to regulate access to sexually explicit online material [9]. Mandatory age verification legislation is one such regulatory mechanism currently gaining global traction. This compels digital content providers to confirm a user’s age before granting access to web pages hosting sexually explicit material [10]. This is commonly achieved through third-party digital services that can authenticate a user’s age through an uploaded government-issued ID, credit card information, or more novel means, such as biometric facial scanning technology [9,11]. In 2017, the United Kingdom became the first jurisdiction to introduce age verification measures for online pornographic content. However, the policy was never enacted and was eventually abandoned 2 years later, following a series of administrative delays [10]. Renewed laws passed in 2023 aim to regulate young people’s access to sexually explicit content, alongside other digital content deemed “harmful” to minors, including material related to self-harm, suicide, and illicit drug use [12]. Age verification laws have also since been implemented in France and Germany. Authorities in both nations are pursuing regulatory action against pornography platforms that have not complied with age verification policies [11,13,14]. Multiple states in the United States have introduced mandatory age verification policies since 2022 [9,15]. In May 2024, the Australian government announced a pilot trial of age verification technology for pornographic content, following the release of the eSafety Commissioner’s Age Verification Roadmap [11,16]. Although these policies remain subject to ongoing debate and legal challenges, they suggest a growing eagerness among policymakers worldwide to introduce age-based restrictions on sexually explicit material.

Age verification policies have attracted criticism from a diverse range of stakeholders. Some digital rights advocates have expressed strong opposition to the recent reforms, raising concerns related to data security, online privacy, and individual freedoms [11,17-19]. In a 2017 survey, just 22% of young respondents supported the introduction of a national filter for online pornographic content [20]. The impact of age verification measures on the adult entertainment industry is another concern, as the complexity and cost of establishing age verification systems present significant challenges for smaller businesses and independent operators within the industry [11]. Furthermore, it is argued that these regulations pose a major barrier for smaller creators and niche communities within the adult entertainment industry (eg, LGBTQIA+ [lesbian, gay, bisexual, transgender, queer, intersex, asexual, and other sexual and gender minority identities] creators and kink or fetish groups), potentially leading to disproportionate censorship and marginalization of diverse sexual expression online [19,21].

Evidence suggests that public support is growing for targeted age-based restrictions on young people’s access to online pornography. For instance, a 2019 survey of New Zealand adolescents revealed that 71% of respondents were in favor of restricting young people’s access [22]. In 2022, the Australian eSafety Commissioner found that 91% of young respondents were in favor of age-based restrictions [23].

There is a need for a more comprehensive understanding of young people’s perspectives on age verification, particularly in a rapidly changing social and legislative environment. This study includes those aged between 15 and 29 years, who represent the age group with the highest level of pornography use in Australia [24]. They are therefore the group most directly impacted by age verification policies, as all attempted use of pornography will require users to verify their age. This age group also includes individuals younger than 18 years, who are the target of these policies, and those who have recently been in this age group but were not subject to age verification. Their perspectives on age verification for pornography and acceptance of these policies will affect the uptake and implementation of interventions. This study seeks to ascertain young people’s attitudes toward age verification for pornography, including which methods of age verification they consider acceptable, and explore associations between these attitudes and participant characteristics.

## Methods

### Study Design and Participant Recruitment

This study used data collected in the 2023 *Sex, Drugs, and Rock ‘n’ Roll* study [25]. It is a cross-sectional online survey conducted annually using REDCap [26]. Young people aged between 15 and 29 years residing in Victoria, Australia, were eligible to participate. Since 2005, this survey has gathered information on young people’s sexual health, mental health, and other behaviors. Participants were recruited through paid online advertisements on social media (ie, Facebook and Instagram), as described by Coombe et al [27]. People who had completed previous *Sex, Drugs, and Rock ‘n’ Roll* surveys were also emailed an invitation to participate. Potential participants clicked a link that directed them to the participant information and consent form. After providing informed consent, they completed the survey. Data were collected between April and June 2023. The reporting of this cross-sectional study followed the STROBE (Strengthening the Reporting of Observational Studies in Epidemiology) guideline (Checklist 1).

### Ethical Considerations

Approval was granted by the Alfred Health Human Research Ethics Committee (326/08). All participants were provided with an information sheet and were required to provide informed consent to commence the survey. Per the ethics approval, parent or guardian consent was not required as minors were deemed mature and capable if they provided informed consent. Participants’ responses were anonymous. The survey took approximately 20 minutes. All participants were eligible to enter a cash prize draw for AUD 250 (AUD $1=US $0.71 as of August 10, 2026) after survey completion. To maintain confidentiality, data that included identifying information for reimbursement purposes were stored separately from the survey responses.

### Measures

For this survey, pornography was defined as visual material (eg, images, photographs, or videos) accessed online that is sexually explicit and designed to cause sexual excitement. Participants were asked about the age at which they first accidentally and intentionally viewed pornography and the frequency of viewing in the last 12 months. Participants were provided with a brief passage offering context regarding contemporary efforts to introduce age verification measures for pornographic content (Multimedia Appendix 1) before being asked, “Do you agree or disagree that everyone should have to prove or verify their age before viewing porn?” Participants were then categorized as supportive of mandatory age verification (strongly agree or agree) or opposed to mandatory age verification (strongly disagree or disagree). Irrespective of their answer, participants were then asked, “If proof of age were required, from what age should people be allowed to view porn?” and “What ways of proving your age would be acceptable?” Then, 4 age verification methods were presented to participants: personal ID upload, credit card verification, parental monitoring, and face ID or facial scanning. Participants could select between 0 and 4 of the options. Finally, participants were invited to share any feedback or concerns about age verification in a free-text response.

Sociodemographic measures relating to sexual and gender identity, age, relationship status, education level, and religiosity were also included. Participants were given the option to select multiple sexual and gender identities; however, for analysis purposes, these were grouped as men, women, and nonbinary or gender-fluid or other, and as either heterosexual or LGBTQIA+. Religiosity was ascertained by asking, “Are you an active member of any religious group (such as a church, temple, mosque, or other group)?—Yes/No,” and 5 options were provided for relationship status: single, in a relationship, open relationship, long-term relationship or married and de facto, and other (specify).

### Data Analysis

Quantitative data were analyzed using Stata 17 software (REDCap Consortium) [28]. Descriptive statistics (frequencies and percentages) were used to describe participant characteristics. Univariable logistic regressions examined associations between each participant characteristic and support for age verification (strongly agree or agree vs strongly disagree or disagree). All participant characteristics were categorical data, except for age of pornography exposure, which was a continuous outcome. “I don’t wish to say” and “I don’t know” responses were treated as missing in analyses. Multivariable logistic regression was then conducted with all variables included in the univariable regressions, except for age of pornography exposure, as this was not asked of participants who had not viewed pornography. Missing data were excluded from regression analysis.

We compared the sociodemographic characteristics of those who provided meaningful text responses to the open-ended question with those who provided blank or nonmeaningful responses (eg, “no” or “N/A”). For comparison purposes, we used chi-square tests for categorical variables and a nonparametric Mann-Whitney *U* test for age, as age was not normally distributed.

For all analyses, *P*<.05 was considered statistically significant. A qualitative analysis of free-text responses was undertaken using a reflexive thematic analysis approach. The reporting of the qualitative component was informed by the SRQR (Standards for Reporting Qualitative Research) guideline (Checklist 2). Responses were exported to Microsoft Excel and read multiple times to facilitate familiarization with the data. Meaningful responses included single words, sentences, or longer passages that contained interpretable content relevant to the topic. Nonmeaningful responses, including blank entries, “no comment” responses, and nonsensical text (eg, “sfkds”), were excluded from analysis.

An initial inductive coding process was undertaken by a single author-researcher (JT) to identify patterns and recurring concepts within the data. Codes were iteratively reviewed and grouped into broader categories, with definitions and exemplar quotes recorded in a codebook table using Microsoft Excel. The preliminary coding framework was reviewed by a second author-researcher (ML) to support interpretation and ensure consistency in categorization. A subsequent deductive phase refined and organized the coding framework, guided by emerging patterns in participant responses. Coding was reviewed again by a second author-researcher (ML) to resolve ambiguities and refine the thematic structure. Codes were then consolidated into 4 overarching themes with corresponding subthemes. Reflexive consideration was given to how the researchers’ perspectives and prior knowledge may have influenced the interpretation of the data.

## Results

### Participant Characteristics

In total, 794 young people completed the survey. Of these, 419 (52.8%) identified as women, 257 (32.4%) as men, and 109 (13.7%) as nonbinary, gender-fluid, or another gender identity. The median age of respondents was 23 (IQR 19‐26) years, and 44.5% (n=353) identified as heterosexual. Further demographic information is available in Table 1.

Most participants had viewed pornographic material (711/794, 89.5% intentionally; 614/794, 77.3% accidentally). The median age at which participants first accidentally viewed pornography was 11 (IQR 9‐12) years, whereas the median age of first intentional viewing was 13 (IQR 12‐15) years. Within the past 12 months, 26.6% (189/711) of participants had viewed pornography weekly, while 21.7% (154/711) had viewed it daily or almost daily.

**Table 1.** Demographic characteristics (N=794).

Demographic characteristics	Values, n (%)
Age group (y)	
15‐17	118 (14.9)
18‐19	93 (11.7)
20‐24	274 (34.5)
25‐29	309 (38.9)
Gender identity	
Men	257 (32.4)
Women	419 (52.8)
Nonbinary or gender-fluid or other	109 (13.7)
I don’t wish to say	9 (1.1)
Sexual identity^a^	
Heterosexual	353 (44.5)
Gay or homosexual or lesbian	93 (11.7)
Bisexual	240 (30.2)
Pansexual	70 (8.8)
Asexual	21 (2.6)
Queer	107 (13.5)
Questioning	32 (4.0)
I don’t label	60 (7.6)
I don’t know	26 (3.3)
I don’t wish to say	2 (0.3)
Other	5 (0.6)
Relationship status	
Single	309 (38.9)
In a relationship	249 (31.4)
Open relationship	32 (4.0)
Long-term relationship^b^	197 (24.8)
I don’t wish to say	7 (0.9)
Completed or currently studying postsecondary education	
Yes	598 (75.3)
No	188 (23.7)
I don’t wish to say	8 (1.0)
Active member of church or religious organization	
No	713 (89.8)
Yes	73 (9.2)
I don’t wish to say	8 (1.0)
Ever viewed pornography intentionally	
Yes	711 (89.5)
No	68 (8.6)
I don’t know	3 (0.4)
I don’t wish to say	12 (1.5)
Ever viewed pornography accidentally	
Yes	614 (77.3)
No	134 (16.9)
I don’t know	1 (0.1)
I don’t wish to say	45 (5.7)
Pornography viewing frequency in the previous 12 months^c^	
Never	66 (9.3)
Less than monthly	172 (24.2)
Monthly	126 (17.7)
Weekly	189 (26.6)
Daily or almost daily	154 (21.7)
I don’t wish to say	4 (0.5)

a Participants could select multiple sexual identities.

b Includes engaged, married, and de facto.

c Among those who had ever viewed pornography intentionally (n=711).

### Support for Mandatory Age Verification

Of 794 participants, 33 (4.2%) did not answer this question. Of the remaining participants (n=761), most supported the notion of mandatory age verification, with 60.3% (459/761) either agreeing (253/761, 33.2%) or strongly agreeing (206/761, 27.1%) with the statement, “Everyone should have to prove or verify their age before viewing online pornography,” and 39.7% (302/761) either disagreeing (207/761, 27.2%) or strongly disagreeing (95/761, 12.5%). In multivariable logistic regression, those identifying as women, aged 18 to 24, or actively religious were more likely to support age verification, whereas those identifying as LGBTQIA+ and viewing pornography at least weekly were less likely to support it (Table 2). An older age at first intentional viewing of pornography was also associated with higher support for age verification.

The median age at which participants thought viewing pornography should be permitted was 16 (IQR 15‐18) years (Multimedia Appendix 1). Of the 794 participants, only 2.9% (n=23) believed that online pornography should be restricted for all ages, while 4.9% (n=39) indicated that pornography should be freely available regardless of age.

**Table 2.** Sociodemographic correlates of support for mandatory age verification^a^.

Characteristics	n	Support, n (%)	Oppose, n (%)	Univariable, OR^b^ (95% CI)	Multivariable, aOR^c^ (95% CI)
Total, N	761	459 (60.3)	302 (39.7)	—^d^	—
Gender					
Men^e^	247	110 (44.5)	137 (55.5)	1.00	1.00
Women	408	299 (73.3)	109 (26.7)	3.42^f^ (2.45-4.77**)**	2.82 (1.83-4.36)
Nonbinary or gender-fluid or other	97	47 (48.5)	50 (51.5)	1.17 (0.73-1.87)	1.37 (0.78-2.43)
Missing	9	3 (33.3)	6 (66.7)	—	—
Age group (y)					
15-17^e^	108	61 (56.5)	47 (43.5)	1.00	1.00
18­-19	88	64 (72.7)	24 (27.3)	2.05^f^ (1.12-3.76)	3.18^f^ (1.61-6.29)
20-24	266	160 (60.2)	106 (39.8)	1.16 (0.74-1.83)	1.75^f^ (1.04-2.96)
25-29	299	174 (58.2)	125 (41.8)	1.07 (0.69-1.67)	1.31 (0.77-2.22)
Sexual identity					
Heterosexual^e^	344	229 (66.6)	115 (33.4)	1.00	1.00
LGBTQIA+^g^	416	229 (55.0)	187 (45.0)	0.61^f^ (0.46-0.83)	0.67^f^ (0.46-0.96)
Missing	1	1 (100)	0 (0)	—	—
Actively religious					
No^e^	683	400 (58.6)	283 (41.4)	1.00	1.00
Yes	71	56 (78.9)	15 (21.1)	2.64^f^ (1.46-4.76)	2.37^f^ (1.23-4.59)
Missing	7	3 (42.9)	4 (57.1)	—	—
Relationship status					
Single^e^	298	170 (57.0)	128 (43.0)	1.00	1.00
In a relationship	239	151 (63.2)	88 (36.8)	1.29 (0.91-1.83)	1.04 (0.70-1.54)
Open relationship	28	9 (32.1)	19 (67.9)	0.36^f^ (0.16-0.81)	0.5 (0.20-1.22)
Long-term relationship^h^	191	125 (65.4)	66 (34.6)	1.43 (0.98-2.08)	1.07 (0.69-1.66)
Missing	5	4 (80)	1 (20)	—	—
Frequency of viewing pornography^i^					
Never^e^	131	102 (77.9)	29 (22.1)	1.00	1.00
Less than monthly	163	114 (69.9)	49 (30.1)	0.66 (0.39-1.12)	0.75 (0.42-1.33)
Monthly	120	79 (65.8)	41 (34.2)	0.55^f^ (0.31-0.96)	0.63 (0.34-1.14)
Weekly	182	90 (49.5)	92 (50.5)	0.28^f^ (0.17-0.46)	0.42^f^ (0.24-0.75)
Daily or almost daily	148	64 (43.2)	84 (56.8)	0.22^f^ (0.13-0.37)	0.45^f^ (0.23-0.86)
Missing	17	10 (58.8)	7 (41.2)	—	—
Age of first intentional pornography viewing^j^ (n=682)	—	—	—	1.13^f^ (1.07-1.20)	—
Age of first accidental pornography viewing^k^ (n=591)	—	—	—	1.04 (0.96-1.13)	—

a “I don’t wish to say” or “I don’t know” responses were not included.

b OR: odds ratio.

c aOR: adjusted odds ratio.

d Not applicable.

e Reference category.

f Statistically significant odds ratios (*P*<.05).

g LGBTQIA+: lesbian, gay, bisexual, transgender, queer, intersex, asexual, and other sexual and gender minority identities.

h Includes engaged, married, and de facto.

i Over the past 12 months.

j This variable was assessed only among participants who had ever intentionally viewed pornography (n=682) and was analyzed as a continuous variable.

k This variable was assessed only among participants who had ever accidentally viewed pornography (n=591) and was analyzed as a continuous variable.

### Acceptance of Age Verification Processes

Of the 4 proposed age verification methods, none was supported by a majority of participants. Uploading ID documents (such as a driver’s license or passport) had the highest level of acceptance at 49.5% (377/761), followed by the use of credit card information (216/761, 28.4%) and parental monitoring (137/761, 18.0%). Facial scanning or Face ID was the least acceptable form of age verification, supported by only 17.0% (129/761) of participants.

### Open-Ended Responses

Approximately one-third of participants (257/761, 33.8%) provided a meaningful free-text response. The average response length for meaningful responses was 40.8 (SD 43.6) words. Participants who provided meaningful responses differed significantly from those who did not with respect to age, level of education, and sexual identity (Table 3).

We generated 4 themes to showcase participants’ feedback and concerns about age verification. Themes included (1) privacy and security considerations, (2) inefficacy and workarounds, (3) impacts on young people’s sexual development, and (4) alternative approaches. There were no clear patterns in responses across different demographic groups, with all themes being represented by people of different ages, genders, and sexualities.

**Table 3.** Sociodemographic characteristics among participants who provided meaningful and nonmeaningful responses to the open-text question (n=761)^a^.

Characteristics	Meaningful response	Nonmeaningful response	*P* value
Age (y), median (IQR)	24.7 (21.2‐27.6)	22.7 (19.5-26.5)	<.001^a^
Age groups (y), n/N (%)			.005^b^
15‐19	49/211 (23.2)	162/211 (76.8)	
20‐24	89/274 (32.5)	185/274 (67.5)	
25‐29	118/309 (38.2)	191/309 (61.8)	
Gender^c^			.14^b^
Women	130/419 (31.0)	289/419 (69.0)	
Men	82/257 (31.9)	175/257 (68.1)	
Nonbinary and other genders	41/109 (37.6)	68/109 (62.4)	
Sexual identity^c^			<.001^b^
Heterosexual	90/344 (26.2)	254/344 (73.8)	
LGBTQIA+^d^	154/416 (37.0)	262/416 (63.0)	
Level of education^c^			.02^b^
High school or lower	42/178 (23.6)	136/178 (76.4)	
Postsecondary education	201/577 (34.8)	376/577 (65.2)	

a *P* value computed using the Mann-Whitney *U* test for continuous, nonnormally distributed variables.

b *P* value computed using the chi-square test for categorical variables.

c Variable categories were collapsed for analysis. Missing data were excluded.

d LGBTQIA+: lesbian, gay, bisexual, transgender, queer, intersex, asexual, and other sexual and gender minority identities.

### Privacy and Security Considerations

User privacy and data security were the most frequently reported issues for participants, comprising more than half (149/257, 58.0%) of free-text responses. Participants were concerned about the highly sensitive nature of the data required for each age verification strategy (ie, credit card details, personal identity documents, and biometric information) and how this personal data would be securely managed. Respondents were resistant to sharing their personal information to access pornographic content, believing it to be highly vulnerable to theft by online hackers. Few participants trusted third-party websites to protect their personal information, whereas others raised concerns about the potential for fake pornography websites to be created with the intention of stealing or harvesting user information.

*It provides more opportunities for personal information to be hacked/stolen/distributed. I don’t think porn sites are exactly known for their reliability or security.*

[Heterosexual woman, 25 years]

*Some scam websites that replicate these porn websites could appear and could steal personal information from vulnerable children.*

[Bisexual/questioning woman, 15 years]

Other respondents discussed the loss of user anonymity and expressed concern that their pornographic preferences and habits could easily be linked to their personal identity. It was suggested that this would result in various forms of exploitation, including blackmail, doxing, and identity theft.

*I don’t want my legal identity tied to my sexual preferences. I don’t want porn studios to have access to my legal info/identity, and I don’t want to have any bureaucracy attached to my porn consumption.*

[Bisexual/queer woman, 25 years]

*I think it’s necessary, but I don’t have any faith in organisations to keep that information secure and [I] am worried of the ramifications of a data leak on individuals’ reputations.*

[Bisexual nonbinary, 24 years]

Of 257 responses, 37 (14.4%) were specifically concerned with the role of the government in facilitating the mandatory age verification process. Participants described the mandatory measures as “authoritative” and “draconian,” while articulating a lack of trust in government authorities to collect and manage user information without infringing on their privacy and personal liberty.

*I do not believe the government would be able to protect people’s data safely and securely, and I do not see how this would stop the issue...creating age verification seems draconian.*

[Bisexual woman, 26 years]

### Inefficacy and Workarounds

Participants expressed doubts regarding the overall efficacy of the proposed age verification strategies. Almost one-third of respondents (75/257, 29.2%) suggested that young people were likely to bypass the age verification measures with relative ease by using freely available workarounds, such as virtual private networks (VPNs), or by using another individual’s personal information (ie, a parent’s credit card or email account).

*People will always find a way to get around access restrictions especially online (eg, it might be age restricted in Australia, but a VPN can easily allow access despite Australian regulation*).

[Pansexual man, 25 years]

*It’s really easy to access guardians’ ID cards, credit cards, they could just be lying around. For the “parental monitoring” option, this could lead to children just making their own email/accessing a parent’s one that’s on the same device.*

[Heterosexual woman, 25 years]

Other respondents (4/257, 1.6%) doubted the ability of the proposed strategies to successfully restrict all forms of pornography, including audio, story-based content, and pornography featured on other online platforms.

*Pornography is available on Twitter and Reddit in places you wouldn’t expect and will likely have trouble monitoring.*

[Heterosexual man, 26 years]

Out of 257 participants, 6 (2.3%) contended that age-based restrictions would, in fact, drive pornography “underground,” resulting in a black market for accessible pornographic content. It was hypothesized that much of this content would consequently be hosted on insecure and dangerous platforms such as the dark web, forcing young people to access pornographic content through more hazardous, unsafe means.

*If children don’t have access to porn due to their age maybe they will go to even more hardcore/dodgy/illegal websites to get access to it.*

[Heterosexual woman, 18 years]

*Kids should be allowed to explore their sexuaity* [sic] *in a safer noninteraction based way. Restricting porn access just means they will try to obtain experience/material in less safe and/or illegal means, such as meeting strangers, chat rooms, physically bought porn etc.*

[Queer man, 17 years]

### Impacts on Young People’s Sexual Development

Respondents (63/257, 24.5%) raised concerns about the impact of mandatory age verification on the healthy sexual development of young people. Pornography was described as a useful resource for exploring and learning about sex and sexuality, particularly in the absence of adequate sexual health education. It was reasoned that mandatory age verification measures would be detrimental to young people’s sexual development because they would deny them the ability to access this source of information during puberty and other formative years.

*Teenagers should be able to use pornography as a healthy/safe way of exploring their sexuality without having to get parents involved.*

[Bisexual woman, 28 years]

*Viewing porn can be an amazing way to discover yourself and your sexuality which is why everybody should see it at least once the main problem with youth viewing porn is that they try to take from it or mimic it when none of it is realistic or feels good. If there was a way to make sure that it was being viewed healthily then there is no problem with it.*

[Bisexual man, 15 years]

Of the 257 responses, 8 (3.1%) specifically highlighted the potential impacts on LGBTQIA+ young people, who may use pornography as a way of exploring and understanding their sexual preferences and identity. It was further suggested that the proposed age verification methods, especially parental monitoring, could result in young people being prematurely outed or caught accessing LGBTQIA+ content.

*Notifying parents that their child is trying to access porn will out closeted LGBTQIA+ teens to their parents and put those young people into dangerous situations. Parents making sure their kids are browsing the web in safe and age-appropriate ways is very different to your dad getting a text saying, “your child is trying to watch gay porn right now.” Parents should not have control or veto powers over the sexuality of their children.*

[Queer/bisexual nonbinary, 28 years]

Other participants (27/257, 10.5%) perceived mandatory age verification as a necessary measure for protecting young people from some of the harmful sexual themes commonly embedded within mainstream pornographic content. Participants were particularly concerned about materials deemed misogynistic and violent, and how exposure to such content would influence young people’s emerging sexual attitudes and behaviors.

*My concern with minors viewing pornography is that mainstream pornography is generally misogynistic and violent. It exposes minors to very harmful stereotypes, attitudes, and expectations in relation to what sex normally looks like*.

[Heterosexual woman, 23 years]

### Alternative Approaches

Participants also gave suggestions for alternative and supplemental strategies. Of the 257 open-text responses, 42 (16.3%) responses related to this theme, and 34 (13.2%) responses called for improved sexual health education and emphasized the need for more critical and comprehensive teaching about pornography alongside other broad sexual health themes (ie, consent, relationships, and sexuality).

*We need to educate teenagers, especially boys, on what healthy, safe sex is and the difference between fantasy porn and reality. Blanket bans on them watching porn won’t work and is not the solution.*

[Heterosexual man, 28 years]

Other responses (23/257, 8.9%) encouraged greater access to, and support for, pornography that is more ethical, alongside widespread industry reform and regulation.

*I believe it should be more educational before viewing example being, clearly stating that it’s a scripted scenario, fake and should not be an expectation for real interactions with others. But I think it is a good way for younger people to explore what they’re interested in while being in their own safe and comfortable environment.*

[Woman, does not label sexuality, 17 years]

Some respondents (5/257, 1.9%), however, suggested that parents remain primarily responsible for managing their children’s internet use, and that age-appropriate conversations were the preferred approach to protecting young people from pornography-based harms. Only a small number of participants (3/257, 1.2%) described existing preventative measures (such as family-based internet filters or self-declared age verification) as sufficient to minimize youth exposure to sexually explicit content.

## Discussion

### Summary of Findings

Policies with widespread acceptance are more likely to be successful [29,30]. This study aimed to understand young people’s attitudes toward age verification, including which methods of age verification were seen as acceptable, and to explore associations between these attitudes and participant characteristics. Our findings indicate that most young people broadly support the introduction of age-based restrictions for online pornographic content. However, participants remained largely unconvinced by the proposed age verification strategies, with none of the 4 methods presented being deemed acceptable by a majority. Those identifying as women, aged 18 to 19 years, actively religious, and with an older age at first intentional viewing pornography were more likely to support age verification, whereas those identifying as LGBTQIA+, in an open relationship, and viewing pornography monthly or more often were less likely to support age verification.

### Interpretation and Implications of Findings

Responses suggest that privacy concerns and the perceived inefficacy of age verification technology reduced their acceptability. These findings closely mirror those of other incipient studies [20,22,23,31,32], including a recently published survey by the Australian eSafety Commissioner [23]. While a similar level of support for age verification on pornographic websites was observed among the cohort of young people (59%), 91% of respondents expressed concern about the specific age verification strategies discussed, and 1 in 5 (20%) were unsure of how to provide proof of age [23]. Apprehensions surrounding data security, personal privacy, and technological efficacy once again featured prominently within this group, with similar concerns also recently observed in other international cohorts [31]. Privacy concerns in our cohort were not limited to concerns of data security but extended to questions about institutional legitimacy. Participants expressed skepticism about whether government authorities could be trusted to manage such sensitive information appropriately, particularly given the uniquely identifying nature of sexual behavior data. Research on young people’s data privacy expectations demonstrates that they demand meaningful transparency and control over their personal information and question whether governance bodies can reliably provide this [33]. These findings suggest that the acceptability of age verification policies is contingent not only on the effectiveness of technical data protection measures but also on policymakers’ capacity to build and maintain public trust in government data governance.

Importantly, our study shows that young people are not a homogeneous group, and their diverse experiences with online pornography may shape their perceptions and acceptance of age verification processes in unique ways. Young women, for example, often express dissatisfaction with how they are portrayed in mainstream heterosexual pornography, as it commonly depicts them in a submissive and obedient manner while emphasizing male satisfaction, dominance, and pleasure [33]. Young women have also reported associations between partner exposure to pornography and negative sexual experiences, as well as low self-esteem and body dissatisfaction [34-36]. It is therefore unsurprising that support for age verification was substantially higher among young women in our study (299/408, 73.3%) when compared with men (110/247, 44.5%) or those who identify as nonbinary, gender-fluid, or another gender identity (47/97, 48.5%). Qualitative responses reflected these concerns, with participants describing mainstream pornography as “misogynistic and violent” with concerns of exposure to “harmful stereotypes, attitudes, and expectations.” Actively religious participants similarly indicated elevated support for age verification (56/71, 78.9%) when compared with their nonreligious counterparts (400/683, 58.6%). This is in line with existing evidence suggesting that religious commitment is associated with lower rates of pornography viewership, greater support for sexual abstinence, and censorship of violent and sexual content [37-39].

In contrast, support for age verification was lower among those with frequent pornography consumption (64/148, 43.2% of daily viewers; 102/131, 77.9% of those who had not viewed pornography in the past 12 months), and among participants who identified as LGBTQIA+ (229/416, 55.0%) compared with heterosexual (229/344, 66.6%). For LGBTQIA+ youth in particular, pornography serves as an important resource for exploring and understanding sexual identity and expression during formative years in the absence of affirming sexual health resources [40], with some participants suggesting that restricting access could impede healthy sexual development. This perspective aligns with contemporary frameworks of adolescent sexual health, which prioritize young people’s capacity to make decisions about their sexual development in line with their evolving capacities [41]. Such theories argue that safeguarding measures should promote such development through empowerment rather than restriction and regulation [19,41]. Ultimately, these patterns reflect fundamentally different conceptualizations of pornography as either a source of harm or a developmental resource, suggesting that a one-size-fits-all approach to age verification is unlikely to uniformly serve young people whose relationships with pornography are shaped by different experiences, values, and developmental needs. Effective and acceptable implementation will require careful engagement with young people to understand and respond to these divergent concerns and to ensure policies remain responsive to their varied needs.

LGBTQIA+ young people also face distinct privacy risks that age verification policies may inadvertently exacerbate. Research indicates that LGBTQIA+ young people commonly adapt their use of online technology as a means of self-preservation to avoid harassment or the unwanted disclosure of their sexual identity [41,42]. The perceived anonymity of the internet also helps to alleviate concerns about potential discrimination and allows LGBTQIA+ youth to explore their sexual and gender expression without fear of stigmatization [43-46]. Participants described concerns that age verification, particularly parental monitoring, could result in the unwanted disclosure of sexual identity, with some warning that it could “out closeted LGBTQIA+ teens to their parents” and put them in “dangerous situations.” These risks highlight a critical policy challenge whereby protective measures that remove anonymity may paradoxically harm the young people they seek to protect, further undermining the acceptability and legitimacy of such strategies among young people.

Finally, alternative and supplemental strategies to age verification featured prominently in participant responses. Notably, many of the described alternative strategies embodied key principles of harm minimization, suggesting a potential rejection of the regulatory and prohibitive nature of age verification measures. For example, the inclusion of pornographic-related information as part of a broader and more comprehensive pornography literacy and sexual health education program was a commonly suggested strategy among qualitative responses. These programs seek to facilitate open discussions and improved understandings of crucial sexual health themes, including pornography, consent, pleasure, and the complexities of real-life sexual activities [20,47]. Such initiatives seek to equip young people with the skills to critically assess key messages in pornographic material, helping them to make safer and more informed sexual health decisions [47,48]. Although simple technological preventions may still offer effective and appropriate protections for younger children, particularly against accidental and unwanted exposure, for older adolescents and LGBTQIA+ young people who are intentionally seeking such content, these education and literacy programs are likely to provide a more realistic and acceptable approach to minimizing potential harms.

### Implications for Public Health and Policymakers

Grelle and Hofmann [29] note 4 components of policy that affect its acceptability: effectiveness, intrusiveness, fairness, and transparency. The effectiveness of age verification was perceived as poor in our study, with many participants noting easy ways to get around restrictions. Age verification is a highly intrusive intervention, and in concordance with other studies, participants expressed a preference for less intrusive harm minimization interventions such as education [49]. Fairness often reflects perceived impact of the policy on oneself in comparison to others; we found that those most likely to be impacted by the policy—those who frequently view pornography—were more opposed to it. As described earlier, women and LGBTQIA+ participants are also differentially affected by pornography and age verification and expressed corresponding views. Finally, relating to transparency, we noted concerns about implementation methods. Studies have also noted that it is common for people to accept the overall concept of a policy (as shown in our study by majority support of age verification) while rejecting specific methods of its implementation (as shown by minority support for individual methods) [30].

It is imperative that governments and policymakers remain prudent amidst a rapidly evolving legislative and technological landscape. The concerns held by young people warrant caution, with substantial efforts needed to first address these apprehensions and rectify potential misconceptions. Personal privacy and data security, for example, were crucial concerns for our participants. Many expressed fears over the potential misuse or exploitation of their personal information, including the possibility that their identity would be linked to the specific content they accessed. Regulators must therefore actively explore and pursue age verification processes that protect user privacy by limiting the collection of essential personal information while ensuring transparency in the data collection process to minimize user anxieties. Although these approaches were not assessed in this survey, novel age verification methods may offer solutions that address some of these concerns. For instance, age verification prototypes developed in France operate on the principle of “double anonymity” and use dual third-party verification services to maintain data confidentiality [50]. This process ensures that no single entity has access to both the personal information of the user and their browsing data, thereby maximizing privacy preservation and limiting the opportunity for data linkage and exploitation. Similarly, Spanish authorities have recently designed multiple prototypes which allow for a localized age verification process contained entirely on the user’s device [51], which may offer similarly acceptable age verification solutions for young people. Future research could consider a broader range of policy and technology options than those explored in this study.

In the open-ended responses, participants pointed out ways to bypass verification methods, such as using parents’ credit cards and identification. These doubts about verification efficacy are valid when considering other research. For example, Stardust et al [19] found that face-scanning technologies are “not even close to being able to reliably distinguish between people who are under and over eighteen years old.”

Our findings underscore the diversity and complexity of young people’s relationships with pornography. Recognizing this and understanding its implications for age verification initiatives are crucial to ensure the effective development of future strategies. Implementing an age verification policy framework should be nuanced and user-centered, fostering understanding of and trust in the procedures. Co-design and community consultation with young people should be prioritized.

### Limitations

Participants in our study were predominantly women, with a relatively high proportion identifying as LGBTQIA+. Most respondents were also educated at a postsecondary level and were located in metropolitan areas, thereby limiting the generalizability of our findings to the wider Australian young population. Participants who were older, had higher education, and identified as LGBTQIA+ were also more likely to provide open-ended responses; therefore, these data are more representative of these demographic categories. As our study shows, these factors are related to acceptance of pornography and age verification, so the results should be interpreted with caution.

Our findings also exclusively pertain to the 4 age verification strategies selected by the research team. We recognize that other mechanisms may be worthy of further exploration, particularly given the rapidly developing nature of age verification technologies. The descriptions of the selected age verification strategies were also kept deliberately brief to facilitate broader discussions of age verification processes. We recognize that participants’ perspectives may change with supplementary information. Qualitative findings were only derived from the 257 brief free-text responses received, representing 32.4% of all survey participants. In-depth qualitative research is needed to better understand young people’s perspectives on the proposed age verification strategies. Further research should also seek the perspectives of other key stakeholders, including parents, teachers, policymakers, and adult entertainment professionals, to gain a more holistic understanding.

### Conclusions

As age verification policies continue to gain global traction, it is imperative that policymakers first understand how these technological measures are perceived by, and how they impact, their potential users. Although our findings suggest there is conceptual support for age verification measures among this sample of participants, none of the proposed age verification strategies were widely accepted. Participants expressed strong concerns regarding user privacy and data security, with many fearful that their personal information could be compromised or exploited. There were also contrasting opinions on how mandatory age verification may affect young people’s sexual development, with some participants worried it would hinder sexual exploration, particularly for LGBTQIA+ young people, whereas others viewed it as crucial for protecting against harmful sexual themes. Finally, many participants doubted the overall effectiveness of these technologies in preventing the intentional viewing of pornography and emphasized less regulatory strategies, such as improved sexual health education, as more suitable alternatives.

## Supplementary material

10.2196/79622 Multimedia Appendix 1 Survey context provided to participants and distribution of responses.

10.2196/79622 Checklist 1 STROBE checklist.

10.2196/79622 Checklist 2 SRQR checklist.

## Abbreviations

LGBTQIA+: lesbian, gay, bisexual, transgender, queer, intersex, asexual, and other sexual and gender minority identities
OR: odds ratio
SRQR: Standards for Reporting Qualitative Research
STROBE: Strengthening the Reporting of Observational Studies in Epidemiology
VPN: virtual private network
