# Supplementary material for: Young People’s Perspectives on Age Verification for Online Pornography in Australia: Cross-Sectional Online Survey
Source: J Med Internet Res. 2026 Aug 18;28:e79622. doi: 10.2196/79622 (PMC13484816; doi:10.2196/79622)
Supplement: Supplementary file 3 [file jmir-v28-e79622-s001.docx]

# Supplementary Material:

### Appendix 1: Brief context paragraph provided to participants in survey

*There is currently work underway by different Government organisations in Australia to implement age-based restrictions for accessing porn online. This is being done to prevent children from viewing online porn. But it would mean that everyone would need to verify/prove their age before viewing porn. We are interested in what young people think about this.*

### Appendix 2: Figure showing range of responses to the question: “If proof of age was required, from what age should people be allowed to view porn?”

Figure 2: Participant’s opinion on the appropriate age for young people to view pornography (frequency [n]) (n=794)

Frequency

| Age (years) | Freq |
| --- | --- |
| 9 | 1 |
| 10 | 2 |
| 11 | 2 |
| 12 | 15 |
| 13 | 36 |
| 14 | 58 |
| 15 | 67 |
| 16 | 265 |
| 17 | 20 |
| 18 | 191 |
| 19 | 1 |
| 20 | 12 |
| Blocked for all ages | 23 |
| Freely available | 39 |
| I don't know | 57 |
| I don't wish to say | 5 |
